# Extubation Failure and Timing to Tracheostomy in Children Surviving Acute Neurological Injury

**DOI:** 10.3390/children12050586

**Published:** 2025-04-30

**Authors:** Ethan L. Gillett, Sneha Jayadeep, Chary Akmyradov, Salim Aljabari

**Affiliations:** 1 Department of Internal Medicine, College of Medicine, University of Arkansas for Medical Science, Little Rock, AR 72205, USA; sjayadeep@uams.edu (S.J.); saljabari@uams.edu (S.A.); 2 Arkansas Children’s Hospital, Little Rock, AR 72205, USA; akmyradovc@archildrens.org

**Keywords:** extubation, neurologic injury, neurocritical care, tracheostomy, pediatrics

## Abstract

**Background/Objectives**: Critically ill patients with acute neurological injury commonly require intubation. The true incidence of and risk for extubation failure in pediatric patients with an acute neurologic injury is not well reported, making the assessment of these patients for extubation readiness or the need for tracheostomy challenging. This study aims to better delineate the incidence of extubation failure and factors associated with the need for tracheostomy in pediatric patients surviving an acute neurologic injury. **Methods**: We conducted a retrospective cohort study using the Virtual Pediatric System (VPS) database of neonates, infants, children, and adolescents < 18 years of age with a neurological injury requiring intubation from 2012 to 2022. Demographic and clinical variables were compared between subjects that were successfully extubated, those with early tracheostomy placement (≤14 days), and those with late tracheostomy placement (>14 days). **Results**: Of the 38,810 enrolled subjects, 37,661 (97.04%) were successfully extubated, 481 (1.24%) underwent early tracheostomy, and 668 (1.72%) underwent late tracheostomy. The most common etiologies were seizures (60.6%), trauma (20.9%), and intoxication (9.1%). The successfully extubated subjects had a higher median initial GCS score (8 vs. 5 and 4, *p* < 0.001) and fewer extubation attempts (1 vs. 3 and 3, *p* < 0.001) than the tracheostomy cohorts. There was a significant difference in median ICU days between the three groups (2.52 vs. 18.3 vs. 38.3, *p* < 0.001). **Conclusions**: The majority of pediatric patients requiring intubation following an acute neurological injury can be successfully extubated. Among patients requiring a tracheostomy, those who received it early had significantly shorter ICU and hospital stays.

## 1. Introduction

Acute neurologic injury is a common reason for admission to a pediatric intensive care unit (PICU) with incidences ranging from 16 to 23% [1,2]. These patients commonly require intubation and mechanical ventilation, often for longer periods of time than other patient populations [3]. Longer durations of mechanical ventilation (typically defined as >21 days) are associated with more complications including ventilator-associated pneumonia, cardiac dysfunction, prolonged immobility, and longer length of ICU stay [4,5]. Conversely, extubation failure requiring reintubation is associated with higher morbidity and mortality [6], making an accurate assessment of extubation readiness crucial for this patient population.

Current recommendations for assessing extubation readiness in children primarily focus on respiratory criteria such as the respiratory rate, oxygen requirement, and lung compliance, while overlooking neurological considerations such as the ability to manage secretions and airway tone, making the decision of when to attempt extubation in these patients challenging [7]. Extubation failure in adult patients with neurologic injury is reported at rates as high as 19% [8]; however, the true incidence of and risk for extubation failure in pediatric patients with an acute neurologic injury is not well reported.

In patients that are unable to be successfully extubated, tracheostomy provides the ability to provide prolonged mechanical ventilation while liberating patients from sedation and promoting earlier mobility and reducing ICU length of stay [9]. While the benefits of tracheostomy in patients with an acute neurologic injury have been reported, the timing of when to best perform this procedure remains controversial, especially in the pediatric population. Providers must also weigh the benefits of tracheostomy and long-term ventilation against the potential risks, such as infections, surgical complications, increased financial, and social strains on families caring for a patient with a tracheostomy, making these decisions all the more challenging.

Tracheostomy in critically ill patients who fail to disconnect from mechanical ventilation or are predicted to fail from mechanical ventilation is a common practice in the adult ICU, with a large body of evidence. It allows for an earlier reduction or cessation of sedative medications, a reduction in oropharyngeal and laryngeal trauma, earlier rehabilitation and ambulation, and improved secretion clearance, which may account for the observed lower frequency of complications [10,11]. It is commonly conducted early in adult critical illness, although the definition of early tracheostomy is still debatable [12]. The practice of tracheostomy in pediatric critical illness is not as standard as in adult medicine and varies quite a bit [13,14]. The literature in this area is scarce.

This study aims to better delineate the incidence of extubation failure and factors associated with the need for tracheostomy in pediatric patients surviving an acute neurologic injury. Secondary aims were to compare the outcomes in pediatric neurocritical care patients that underwent early (<14 days) vs. late tracheostomy (>14 days).

## 2. Materials and Methods

The study design was approved by the Institutional Review Board for the University of Arkansas for Medical Sciences (project #275136). For this type of retrospective study, it was deemed to not be human subject research; therefore, formal consent was not required. To obtain our patient population, we used a large multi-institutional PICU database, the Virtual Pediatric Systems (VPS) database.

The VPS database is an online database that uses standardized clinical definitions, data quality control, and data analysis through the collection of prospective observational data of PICU admissions from more than 150 hospitals. Data validation is performed by both individual sites and in the VPS with an interrater reliability of greater than 95% [15].

In the VPS database, patients are de-identified, and each patient is assigned a “primary diagnosis”, defined as the principal reason for the patient’s admission to the PICU or the condition that resulted in the need for critical care intervention. It is determined based on physician documentation at PICU discharge. Neurocritical care diagnoses were drawn from a prespecified list that included various causes of acute brain injury, seizures and status epilepticus, spinal cord pathology, and neurosurgical disorders [16].

In this retrospective cohort analysis, we analyzed patient data submitted to the VPS database during the time period of January 2012 to December 2022. Our study population consisted of children from birth to ≤18 years admitted to the PICU with a primary neurological or neurosurgical diagnosis who required intubation and mechanical ventilation. We excluded patients who died or those who had congenital malformations, spinal cord abnormalities, and neuromuscular disease. In cases with multiple relevant neurologic diagnoses, the primary associated diagnosis was used.

We divided this patient population into three cohorts for comparison, i.e., cohort 1 consisting of patients who were successfully extubated, cohort 2 consisting of patients that required tracheostomy placement but in ≤14 days from admission (early tracheostomy cohort), and cohort 3 consisting of patents that required tracheostomy placement but >14 days from admission (late tracheostomy cohort).

Data used for our assessment included demographic characteristics including age, sex, race, and weight, primary neurological/neurosurgical diagnoses, and admission Glasgow Coma Scale (GCS), Pediatric Index of Mortality 3 (PIM3), and Pediatric Risk of Mortality 3 (PRISM3), baseline pediatric cerebral performance category (PCPC) and pediatric overall performance category (POPC) scores. Outcome measures included the number of extubations per patient, discharge PCPC and POPC scores, and ICU and hospital lengths of stay.

### Statistical Analysis

Descriptive statistics were used to summarize the data, with categorical variables presented as frequency counts and percentages and continuous variables summarized using means and standard deviations or medians and interquartile ranges, as appropriate. Group comparisons in univariate analyses were conducted using the Chi-square test or Fisher’s exact test for categorical variables and either the independent *t*-test or Mann–Whitney U test for continuous variables, depending on normality as assessed via the Shapiro–Wilk test.

To identify key characteristics distinguishing cohort groups, multiple logistic regression was performed. Given the presence of overdispersion, negative binomial regression was used in univariate analyses to model ICU length of stay and hospital length of stay. Collinearity among covariates was evaluated using cluster analysis and correlation coefficients. A zero-truncated negative binomial regression model was employed for multivariable regression to predict these outcome variables.

All statistical analyses were conducted using SAS 9.4 (SAS Institute, Cary, NC, USA). Statistical tests were two-sided, with a significance threshold of *p* < 0.05.

## 3. Results

A total of 38,810 enrolled subjects met criteria and were analyzed. The majority of the population was aged 29 days to <2 years (30.75%), white (48.03%), and male (57.98%). The demographic characteristics are shown in Table 1 with admission characteristics displayed in Table 2. The most common diagnosis was “seizures” (60.62%), followed by “trauma” (20.93%) and “neurovascular” (16.48%). The majority of patients had a normal baseline PCPC score (68.12%) and a “good overall performance” POPC score (59.97%). There was a mean PIM score of −3.91, a median PRISM3 score of 3 (IQR 0, 7), and a median GCS of 8 (IQR 6, 13) on admission. The median ICU length of stay (LOS) was 2.61 (IQR 1.18, 7.67) days with a median hospital LOS of 6.96 (IQR 2.70, 19.66). The majority of patients were discharged with a normal PCPC score (45.87%) and a POPC score of “good overall performance” (31.25%).

### 3.1. Successful Extubation Characteristics

Of the study population, 37,661 (97.04%) were successfully extubated prior to discharge. They underwent a median of one extubation attempts (IQR 1.1). The majority were aged 29 days to <2 years (30.93%). The most common associated diagnosis was “seizure” (61.2%). There was a mean PIM score of −3.95, a median PRISM3 score of 3 (IQR 0.7), and a median GCS score of 8 (IQR 6.13) on admission. The median ICU LOS was 2.51 (IQR 1.13, 6.95) days with a median hospital LOS of 6.60 (IQR 2.66, 17.96). Most patients had a normal PCPC score (46.91%) and a POPC score of “good overall performance” (32.02%) at the time of discharge (Table 3).

### 3.2. Early Tracheostomy Characteristics

A total of 481 (1.24%) subjects received a tracheostomy within 14 days of admission. The earliest tracheostomy was performed at 5 days. They underwent a median of three extubation attempts (IQR 2, 4). The most common age was 12 to <18 years (33.89%). The most common diagnoses in this group were “seizures” (37.63%) and “trauma” (31.6%). There was a mean PIM score of −3.22, a median PRISM3 score of 6 (IQR 1, 12), and a median GCS score of 5 (IQR 3, 8.5) on admission. The median ICU LOS was 18.30 (12.58, 24.99) days with a median hospital LOS of 33.27 (IQR 20.77, 57.63). Most patients had a “severe disability” PCPC score (44.94%) and a POPC score of “severe overall disability” (46.07%) at the time of discharge.

### 3.3. Late Tracheostomy Characteristics

There were 668 (1.72%) subjects that received a tracheostomy after 14 days. They underwent a median of three extubation attempts (IQR 2, 4). Most were aged 12 to <18 years (30.99%) with the most common diagnoses being “seizure” (44.31%) and “trauma” (33.53%). There was a mean PIM score of −2.56, a median PRISM3 score of 8 (IQR 3,16), and a median GCS of 4 (IQR 3, 8) on admission. The median ICU LOS was 38.22 (IQR 28.71, 57.46) days with a median hospital LOS of 70.41 (IQR 47.40, 115.88). Most patients had a “severe disability” PCPC score (45.11%) and a POPC score of “severe overall disability” (54.48%) at the time of discharge.

### 3.4. Group Comparison

**Figure 1:**
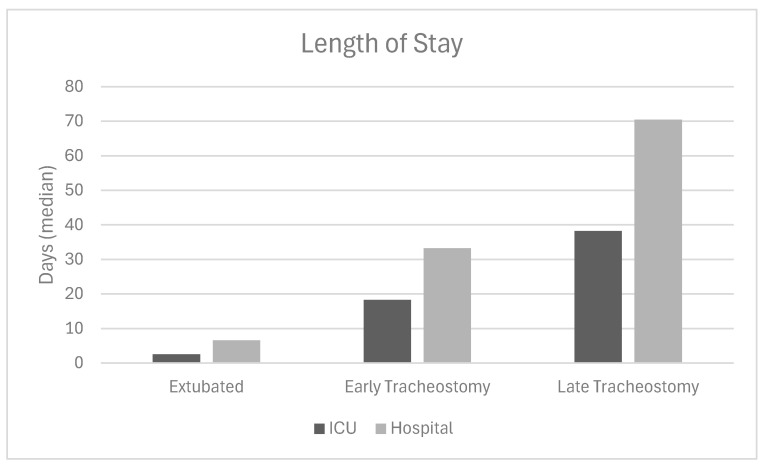
Length of stay for patients with an acute neurologic injury. ICU: intensive care unit; Early tracheostomy: <14 days; Late tracheostomy: >14 days.

The successfully extubated subjects had a higher median initial GCS score (8 vs. 5 and 4, *p* < 0.001) and fewer extubation attempts (1 vs. 3 and 3, *p* < 0.001) than the tracheostomy cohorts. There was a significant difference in median ICU days between the three groups (2.52 vs. 18.3 vs. 38.3, *p* < 0.001) and hospital LOS (6.60 vs. 33.27 vs. 70.41, *p* < 0.001, Figure 1). Between the two groups that received tracheostomy, there was no significant difference in the etiology of the neurologic injury (*p* = 0.125). Of the 34,751 patients who were successfully extubated, fewer than 10% required more than one extubation attempt, and less than 1% needed more than two attempts.

## 4. Discussion

Neurologic-based variables for extubation failure and the need for and practices surrounding tracheostomy following pediatric neurocritical care illness remain largely unstudied. We report the extubation failure rate in a large sample of children who survived a primarily neurological critical illness. We also report on the likelihood of successful extubation once the first attempt of extubation failed in this patient population. And finally, we compare the outcome when early tracheostomy is performed compared to late tracheostomy. To our knowledge, this is the first study to investigate those questions in this patient population.

The majority of patients in our study (97.04%) were successfully extubated, with a median of one extubation attempt. These patients had higher initial Glasgow Coma Scale (GCS) scores, shorter ICU and hospital lengths of stay (LOS), and better functional outcomes at discharge compared to those who required tracheostomy. In contrast, patients who underwent tracheostomy, whether early (≤14 days) or late (>14 days), had more severe initial neurological injuries, as evidenced by lower GCS scores, higher Pediatric Risk of Mortality (PRISM3) scores, trauma as the primary diagnosis, older age, and a greater number of extubation attempts. We were able to identify several factors associated with successful extubation in this patient population. Our data, while very robust in size and with the strength of being multicenter in origin, are limited to what is recorded in the VPS registry. A prospective study, with neurologically focused data elements, might be able to provide the bases for a predictive model for predicting successful extubation in acute brain injury patients [17,18].

Our findings highlight the significant decline in successful extubation likelihood after multiple failed attempts in patients intubated for primary neurological illness. Specifically, our data suggest that patients who fail their first extubation attempt have less than a 10% chance of success on the second attempt. Furthermore, those who fail two consecutive attempts have less than a 1% chance of success on the third. These results, derived from a large sample, provide valuable insights for clinicians and families making critical decisions about further extubation attempts or considering tracheostomy. Understanding these probabilities may help guide discussions on the risks and benefits of continued extubation efforts versus transitioning to a more definitive airway management strategy.

The decision of if and when to proceed with tracheostomy in pediatric neurocritical care patients is challenging as there are no consensus guidelines for this, and clinicians may take a more cautious approach to tracheostomy in these patients as many experience significant neurological improvement during their hospital stay. Commonly, those children who eventually require tracheostomy placement undergo multiple cycles of extubation and re-intubation which prolong their stay and increase their risk of complications. These patients, in most cases, need to return to sedation and a new ventilatory weaning process, in addition to being more susceptible to complications inherent to mechanical ventilation, such as atelectasis and pneumonia, which can prolong treatment time. We found that a majority of patients who underwent late tracheostomy had ≥4 extubation trials prior to tracheostomy placement.

Our findings support the practice of early tracheostomy when appropriate. The early tracheostomy group had a significantly shorter ICU and hospital LOS (20 days and 37 days less, respectively) compared to the late tracheostomy group, suggesting that earlier intervention may mitigate some of the complications associated with prolonged mechanical ventilation. This is especially the case if the patient has failed two attempts of extubation. Our findings are consistent with adult studies [19] and few small pediatric studies [9,20].

A few small studies with general pediatric ICU patients and pediatric TBI patients have shown promising results. Lee et al. assessed 111 pediatric ICU patients who underwent early vs. late tracheostomy, defined at 14 days, finding that early tracheostomy was associated with a reduced duration of mechanical ventilation and length of ICU and hospital stay [20]. Similarly, in a study of 361 children with severe TBI who underwent tracheostomy placement, Mclaughlin et al. found that early tracheostomy (within 14 days) was associated with fewer ventilator days, a shorter ICU length of stay, a shorter hospital length of stay, and a shorter time to gastrostomy placement [9].

One of the more unexpected findings of our study was the comparable number of subjects in the early tracheostomy group and the late tracheostomy group. This was surprising given the absence of standardized recommendations favoring one approach over the other. However, this balance in sample size ultimately strengthened our comparison, enhancing the reliability and validity of our findings. With both groups being more evenly matched, our analysis was less susceptible to bias, allowing for a more accurate assessment of the potential benefits and risks associated with early versus late tracheostomy. This unexpected yet favorable distribution of subjects provided a more robust foundation for drawing meaningful clinical conclusions.

Future studies aimed at better assessing the risk of extubation failure and the need for tracheostomy, combined with the implementation of early tracheostomy practices, could significantly reduce physiological stress and complications associated with reintubation. This approach may lead to shorter ICU and hospital stays, decreased reliance on sedation and its associated complications, and more timely rehabilitation interventions. It would also be beneficial to obtain follow-up data on patients discharged with a tracheostomy to better inform providers regarding long-term complications or mortality associated with tracheostomy. We consider our work just the beginning of efforts to move the needle in this important and relatively unexplored area of pediatric critical care medicine. Pediatric neurocritical illnesses account for nearly a quarter of PICU admissions [21].

This study has several limitations that may influence the results. First, its retrospective design introduces the potential for selection bias and unmeasured confounding variables. Second, the use of a large database, while advantageous for its size and multi-institutional representation, may be incomplete and lack granularity in certain clinical details, such as regional practices, variation in extubation practices between centers, specific reasons for extubation failure, or the exact timing of rehabilitation interventions. Third, primary diagnosis was extrapolated from the data registry and subject to inaccuracies. Finally, the decision for early vs. late tracheostomy is more complex than a physician decision as it involves multiple factors such as when the patient is stable for the procedure and guardian consent and ability to care for a child with a tracheostomy. There were no data available on potential complications incurred following tracheostomy.

## 5. Conclusions

This study indicates that most pediatric patients with an acute neurological injury can be successfully extubated. Admission GCS scores, PRISM3 scores, and diagnosis may help clinicians assess the likelihood of safe extubation in neurocritical care patients. However, for those who fail extubation and require a tracheostomy, performing the tracheostomy procedure earlier may lead to a shorter hospital stay and improved outcomes. Additionally, our findings indicate that the likelihood of successful extubation after two failed attempts in this patient population is approximately 1%.

## Figures and Tables

**Table 1:** Demographics of pediatric patients surviving an acute neurologic injury by ability to successfully extubate.

Demographics	Successful Extubation (N = 37,661, 97.04%)	Early (</= 14 days) Tracheostomy (N = 481, 1.24%)	Late (>14 days) Tracheostomy (N = 668, 1.72%)	Total (N = 38,810)	*p*-Value
**Age**					<0.0001
Adolescent 12 yrs to < 18 yrs	8462 (22.47%)	163 (33.89%)	207 (30.99%)	8832 (22.76%)	
Child 6 yrs to < 12 yrs	7899 (20.97%)	118 (24.53%)	148 (22.16%)	8165 (21.04%)	
Child 2 yrs to < 6 yrs	8961 (23.79%)	100 (20.79%)	111 (16.62%)	9172 (23.63%)	
Infant 29 days to < 2 yrs	11647 (30.93%)	98 (20.37%)	188 (28.14%)	11933 (30.75%)	
Neonate Birth to 29 days	691 (1.83%)	2 (0.42%)	14 (2.1%)	707 (1.82%)	
**Race**					<0.0001
American Indian or Alaska Native	361 (1.06%)	5 (1.17%)	5 (0.81%)	371 (1.05%)	
Asian	517 (1.51%)	5 (1.17%)	14 (2.27%)	536 (1.52%)	
Asian/Indian/Pacific Islander	699 (2.05%)	10 (2.33%)	12 (1.94%)	721 (2.05%)	
Black or African American	6676 (19.53%)	91 (21.21%)	174 (28.16%)	6941 (19.7%)	
Hispanic or Latino	5489 (16.06%)	73 (17.02%)	91 (14.72%)	5653 (16.05%)	
Native Hawaiian or Other Pacific Islander	82 (0.24%)	0 (0%)	2 (0.32%)	84 (0.24%)	
Other/Mixed	2464 (7.21%)	27 (6.29%)	65 (10.52%)	2556 (7.26%)	
Unspecified	1405 (4.11%)	12 (2.8%)	28 (4.53%)	1445 (4.1%)	
White	16,485 (48.23%)	206 (48.02%)	227 (36.73%)	16,918 (48.03%)	
**Sex at birth**					0.5903
Female	15,842 (42.06%)	196 (40.75%)	270 (40.42%)	16,308 (42.02%)	
Male	21,818 (57.93%)	285 (59.25%)	398 (59.58%)	22,501 (57.98%)	

**Table 2:** Admission characteristics of pediatric patients surviving an acute neurologic injury by ability to successfully extubate. PCPC: pediatric cerebral performance category; POPC: pediatric overall performance category; PIM: Pediatric Index of Mortality; ST. dev: standard deviation; PRISM: Pediatric Risk of Mortality, IQR: interquartile range; GCS: Glasgow Coma Scale.

Admission Characteristics	Successful Extubation (N = 37,661, 97.04%)	Early (<14 days) Tracheostomy (N = 481, 1.24%)	Late (>14 days) Tracheostomy (N = 668, 1.72%)	Total (N = 38,810)	*p*-Value
**Primary Diagnosis**					<0.0001
Neurologic	37,661 (100%)	481 (100%)	668 (100%)	38,810 (100%)	
Seizures	23,051 (61.21%)	181 (37.63%)	296 (44.31%)	23,528 (60.62%)	
Infection	3148 (8.36%)	43 (8.94%)	120 (17.96%)	3311 (8.53%)	
Neurovascular	6141 (16.31%)	93 (19.33%)	161 (24.1%)	6395 (16.48%)	
Trauma	7747 (20.57%)	152 (31.6%)	224 (33.53%)	8123 (20.93%)	
Toxin	3318 (8.81%)	91 (18.92%)	127 (19.01%)	3536 (9.11%)	
**Baseline PCPC Score**					0.0002
Normal	5560 (68.1%)	53 (56.99%)	105 (77.21%)	5718 (68.12%)	
Mild disability	1099 (13.46%)	5 (5.38%)	13 (9.56%)	1117 (13.31%)	
Moderate disability	930 (11.39%)	13 (13.98%)	11 (8.09%)	954 (11.37%)	
Severe disability	570 (6.98%)	21 (22.58%)	6 (4.41%)	597 (7.11%)	
Coma or vegetative state	6 (0.07%)	1 (1.08%)	1 (0.74%)	8 (0.1%)	
**Baseline POPC Score**					<0.0001
Good overall performance	4884 (59.9%)	45 (48.39%)	98 (72.06%)	5027 (59.97%)	
Mild overall disability	1336 (16.38%)	8 (8.6%)	15 (11.03%)	1359 (16.21%)	
Moderate overall disability	1316 (16.14%)	18 (19.35%)	14 (10.29%)	1348 (16.08%)	
Severe overall disability	611 (7.49%)	21 (22.58%)	8 (5.88%)	640 (7.63%)	
Coma or vegetative state	7 (0.09%)	1 (1.08%)	1 (0.74%)	9 (0.11%)	
**PIM, Mean (St. dev)**	−3.95 (1.25)	−3.22 (1.99)	−2.56 (2.28)	−3.91 (1.30)	<0.0001
**PRISM3, Median (IQR)**	3 (0, 7)	6 (1, 12)	8 (3, 16)	3 (0, 7)	<0.0001
**GCS Median (IQR)**	8 (6, 13)	5 (3, 8.5)	4 (3, 8)	8 (6, 13)	<0.0001

**Table 3:** Outcomes of pediatric patients surviving an acute neurologic injury by ability to successfully extubate. LOS: length of stay; PCPC: pediatric cerebral performance category; POPC: pediatric overall performance category; IQR: interquartile range.

Outcomes	Successful Extubation (N = 37,661, 97.04%)	Early (<14 days) Tracheostomy (N = 481, 1.24%)	Late (>14 days) Tracheostomy (N = 668, 1.72%)	Total (N = 38,810)	*p*-Value
**Extubations attempts, Median (IQR)**	1 (1, 1)	3 (2, 4)	3 (2, 4)	1 (1, 1)	<0.0001
**Patients per Number of Extubation Attempts**					<0.0001
1	34,751 (92.27%)	24 (4.99%)	2 (0.3%)	34,777 (89.61%)	
2	2513 (6.67%)	201 (41.79%)	211 (31.59%)	2925 (7.54%)	
3	321 (0.85%)	124 (25.78%)	185 (27.69%)	630 (1.62%)	
4+	76 (0.2%)	132 (27.44%)	270 (40.42%)	478 (1.23%)	
**ICU LOS, Median (IQR)**	2.51 (1.13, 6.95)	18.30 (12.58, 24.99)	38.22 (28.71, 57.46)	2.61 (1.18, 7.67)	<0.0001
**Hospital LOS, Median (IQR)**	6.60 (2.66, 17.96)	33.27 (20.77, 57.63)	70.41 (47.40, 115.88)	6.96 (2.70, 19.66)	<0.0001
**Discharge PCPC Score**					<0.0001
Normal	3755 (46.91%)	11 (12.36%)	8 (6.02%)	3774 (45.87%)	
Mild disability	1857 (23.2%)	9 (10.11%)	8 (6.02%)	1874 (22.78%)	
Moderate disability	1447 (18.08%)	18 (20.22%)	26 (19.55%)	1491 (18.12%)	
Severe disability	870 (10.87%)	40 (44.94%)	60 (45.11%)	970 (11.79%)	
Coma or vegetative state	76 (0.95%)	11 (12.36%)	31 (23.31%)	118 (1.43%)	
**Discharge POPC Score**					<0.0001
Good overall performance	2561 (32.02%)	5 (5.62%)	3 (2.24%)	2569 (31.25%)	
Mild overall disability	2268 (28.35%)	6 (6.74%)	2 (1.49%)	2276 (27.68%)	
Moderate overall disability	2112 (26.4%)	26 (29.21%)	25 (18.66%)	2163 (26.31%)	
Severe overall disability	977 (12.21%)	41 (46.07%)	73 (54.48%)	1091 (13.27%)	
Coma or vegetative state	81 (1.01%)	11 (12.36%)	31 (23.13%)	123 (1.5%)	

## Data Availability

Data are not publicly available. Please contact the corresponding author to inquire about accessing the deidentified data which could be made available for other investigators.
